# Clinical Cases

**Published:** 1889-03

**Authors:** E. W. Berridge

**Affiliations:** London


### CLINICAL CASES.

E. W. Berridge, M. D., London.

(1) *Lactuca virosa.*—April 24th, 1883.—Mr. T., aged thirty-seven, has suffered since childhood from heart-burn; it is brought on by sherry, whisky, mustard, oatmeal porridge, toast, or even the plainest food. Lately has frequently had *bruised pain at anus. Lactuca virosa*$^{cm}$ (Swan) twice daily for fourteen days.

May 17th.—Writes that the medicine effected wonders with regard to the heart-burn; does not remember to have ever been so entirely free from it. Bruised pain less.

February 11th, 1884.—Writes that he has been very well for some time, but has had more constipation, and sore, bruised pain at anus. *Lactuca virosa*$^{cm}$ (Swan) twice daily for fourteen days.

February 27th.—The medicine proved to have been too frequently repeated, and caused a severe aggravation. He wrote that the constipation was less, but he had been greatly troubled with heart-burn, caused by brown bread or porridge. Anus feels very sore and bruised. I gave no medicine.

March 28th.—Wrote that he was much better; much less heart-burn.

April 17th.—Wrote that there had been no heart-burn lately, and when he wrote again, July, 1887, it had not returned.

(2) *Rhus toxicodendron.*—Mrs. C., aged fifty. October 10th, 1882, sprained her right knee three weeks ago when getting out of a carriage with a high step. After rubbing it with Pond's Extract it improved, but for the last week has been much worse. (Suggestion for Count Mattei, to add to his materia medica *Hamamelis*, under the name of *Anti-rheumatico.*) There is no swelling, but the hollow of right knee is tender, with acute pain there on moving; there is *pain there when knee is in bent position; relieved when it is stretched out straight;* the pain goes down calf; sometimes it shoots from hollow of knee to ankle. Acute pain in right calf on putting right foot to ground in walking, when the heel comes down; less severe if she walks on her toes. Pain in leg is *worse on beginning to walk; relieved by continued motion. Rhus toxicod.*$^{101m}$ (F. C.) thrice daily for six days.

October 17th.—Pain less acute; more of a dull pain in calf, shifting toward heel occasionally. Cannot get in or out of carriage without severe pains. Repeat medicine for six days.

August 13th, 1883.—Knee became quite well in three or four weeks. Now at times has sharp pains in outer side of right knee, then it goes to inner side; sometimes sharp pains in right calf. Pain in right knee on going down-stairs, and sometimes in left. Pain is worse on commencing to walk. At times when walking, right patella seems to move out of place. *Rhus tox.*$^{mm}$ (Fincke) twice daily for eight days.

August 21st, writes that the pain in knee is worse, and the giving way of the patella is very painful. *No medicine.*

September 24th, writes that the symptoms soon ceased; but has now strained the left knee, producing exactly the same symptoms as those of the right knee. *Rhus tox.*$^{mm}$ (Fincke) twice daily for fourteen days.

September 27th, wrote that the knee was much better. She soon recovered, and has had no return of these symptoms up to present time (1888).

In this case, Fincke's millionth potency seems to have acted better and more permanently than Skinner's 101M.

(3) *Sarsaparilla*$^{cm}$ (Fincke) cured swelling of spermatic cords after ungratified sexual excitement. This symptom I have verified in other cases.

(4) *Sulphur.*—The following case was communicated by a patient: September 14th, 1888.—A young lady had had for three days, on blowing nose, a stinging pain beginning in right side of nose and going up to forehead in the middle line; yesterday, at the same time, swelling of a vein in forehead just above nose was observed. For two weeks darting pain in right knee, worse when the right leg is lifted to step, or when it is off the floor while sitting, or if it is too far back in walking, or if she puts it too far back when sitting. She took one dose of *Sulphur*$^{30}$ at three P. M.

September 18th.—Pain and venous swelling on blowing nose quite gone; almost gone on 15th. Pain in knee very slight on 16th; quite gone yesterday.

(5) *Lactic acid.*—January 26th, 1886.—Miss D., knuckle of right forefinger red and painful, aching on touch. *Lactic acid*$^{cm}$ (Fincke) thrice daily.

Took medicine for four days and was well; then pain came on in right great-toe joint, soon passing off.

(6) *Belladonna.*—Mrs. C., aged eighty-seven, November 22d, 1882, complains of paroxysms of darting pains in left lower jaw, *coming and going suddenly;* it has been worse since a mental shock; has been subject to it for thirty or forty years. *Belladonna*$^{cm}$ (F. C.) every three hours for six days.

November 29th.—Neuralgia, less severe and less often. *Belladonna*$^{cm}$ (F. C.) thrice daily for six days.

December 12th.—Neuralgia quite gone. She says the medicine acted wonderfully, *and that it is the first time the result has given her any faith in treatment for it.* She had been under the treatment of the late Drs. Quin, Partridge, and Hibbers, besides two living mongrels, but *Bellad.*$^{cm}$ was the first medicine which ever did real good. She had no return of the neuralgia till April 19th, 1883, when a repetition of the medicine soon cured it. Subsequently she had other attacks, which, at her advanced age, is not surprising, but the same remedy again relieved.

(7) *Spigelia.* Miss S. H., aged forty-four. July 11th, 1888.—For fourteen days has had neuralgia, which increases, and last night was worse than ever. It comes on, *between* 11.15 P. M. and 11.30 P. M., about fifteen or twenty minutes after lying down in bed; beginning in left malar bone extending down face, sometimes to neck, and only on left side. The pain is shooting downward, with burning and throbbing and the part feels swollen. It continues till about 3.15 A. M. or 3.30 A. M., when she gets to sleep. It is worse lying, *better directly she stands up.* It is the same whichever side she lies on. It is temporarily relieved by hard pressure, and by either cold or warm applications. During day left side of face feels as if it had been scorched, but no actual pain; only a throbbing of it. Pain is better after eating.

She had tried to cure herself by "Christian Science," but she found that denying that she was in pain had no effect.* *Spigelia*$^{5m}$ (F. C.) one dose at ten A. M.

July 12th.—The pain came on last night about 11.15 P. M., but not nearly so bad as before; much less throbbing and shooting; it was more a dull pain. Fell into a half sleep soon after 12.30 A. M. Woke at 2.30 A. M., and pain was worse, but not so bad as last night, and got better at 3.30 A. M., soon after which she slept much better than before. This morning some catarrh and cough, which passed off by afternoon.

July 13th.—The last two days have been very cold; was

---

* NOTE.—One of the high priestesses of this new faith is deeply pitted from smallpox, one eye is distorted and disorganized, and she is lame from disease of the bones of one leg. Nevertheless, she proclaims publicly the all-sufficiency of Christian Science, and looks forward to a time when it will overcome death itself. "Physician, heal thyself." I am informed by an Orientalist that whatever in "Christian Science" is true, was known ages ago to the Hindoo *yogis*, and comprehended much more scientifically also.

compelled to have a fire yesterday. Last night went to sleep as when in health, woke a little after three A. M., with throbbing but no shooting; it was not nearly so bad as before, and she did not have to rise; it lasted about forty-five minutes, when she went to sleep, and it did not return.

July 14th.—Yesterday weather was much warmer. Woke at about 3.15 A. M. last night, and left side of face felt stiff and tight, with very slight pain; it was much less than previous night, and lasted only fifteen minutes, and she then slept, and the pain did not return.

July 15th.—Went to bed last night at 10.45 P. M., woke as before at 3.15 A. M., but felt only a little soreness and stiffness of face, which did not keep her awake.

July 16th.—Last night no pain or other symptom. Quite well and has remained so (December, 1888).

(8) *Calcarea carbonica.* April 26th, 1887.—Mrs. C. since April 16th, has had "cramp" in hollow of right knee and right calf, but worse in the knee; she says it is like a dart from hollow of knee to calf, and once extended as far as ankle. It comes on if she bends leg; *must keep it straight when walking.* She can bend leg when lying, but it pains her if she bends it when walking. Yesterday she felt the pain in hollow of knee when stretching leg out. *Calcarea carb.*$^{mm}$ (F. C.) one dose.

May 17th.—Reports that the pain went in a day or two, and did not return.

(9) *Cocculus*$^{cm}$ (Fincke) cured enlargement of liver after accouchement; the indication being that the liver was more painful *after anger* (Lippe's *Repertory*, p. 124).

(10) *Magnesia muriatica.*—Mrs. B., subject to gall-stones, had *burning in right hypochondrium* up to the right scapula; deep breathing on moving right arm, catches her in right scapula; pain in right hypochondrium *on putting right foot forward in walking.* If she reads much, or tries her eyes, the left eye seems to turn inward toward nose. *Magnesia muriatica*$^{200}$ at first every four hours, then twice daily cured; she improved much in two days.

(10) *Sepia.*—The same patient, after seven or eight weeks, had two attacks of shivering, the last at three A. M. This was followed at seven A. M. by pain in liver, afterward going across to stomach and spleen, and round back; liver relieved by lying on it; pain generally *relieved by eructations*, and *she feels it would be better if she could pass flatus* downward. *Sepia*$^{cm}$ (Fincke) every thirty minutes, till better, then every two hours. The pain decreased after first dose, commencing in the spleen. Next day so much better that I stopped the medicine, and she soon recovered.

(11) *Cannabis sativa.*—November, 1882.—C. W. B., aged forty-five, consulted us for progressive locomotor ataxia. Among other symptoms, he had for five years been subject to pains from waist to feet, sharp, lacerating, jumping about, sometimes as *if red-hot pincers* were applied to upper-posterior part of thigh, giving the part a wrench; the pain compels him to hold on to something firmly.

*Cannabis sativa*$^{cm}$ (Fincke), a dose every other day for about eight weeks very considerably improved these pains, with general improvement in his health. (See *Encyclopædia*, symptoms 371, 411.) This symptom of "pinching" seems to be characteristic of *Cannabis*.

(12) *Phosphorus—Calcarea.*—August 8th, 1881, Miss G. complained of constant aching in small of back for three weeks, worse when walking or *lifting;* back always weak. Sleepy at any time, but is unrefreshed on waking in morning. Feels worse in morning. For a day or two has had frequent diarrhœa. Giddy on rising. For some months pain at stomach-pit on leaning forward to write; it is sudden, sharp, moves up to top of central portion of chest and round to cardiac region. *Has studied much*, and the food at the college has not been good. *Phosphorus*$^{cm}$ (F. C.), a dose daily for seven days.

August 17th.—Much better after second dose. Can now lift anything without pain. No pain in back since third dose, and no weakness there. The abnormal sleepiness ceased after third dose, and she sleeps well and naturally at night. No giddiness. Less diarrhœa. Stomach-pain returned of itself yesterday when leaning back in a chair. *No medicine.*

February 17th, 1883.—Reports that the pain in back returned within the same year, but passed away without treatment, and has not returned. For a month has had giddiness during school-work as if she did not know for ten minutes where she was. For over a year, has had pain in cardiac region when lying on left side at night, sometimes also in the daytime. For three or four weeks *cannot sleep from multiplicity of thoughts. Calcarea carb*$^{cm}$ (F. C.) once daily for seven days.

February 24th.—No giddiness for last four days; no pain in chest for last two nights. Sleep improving. Feels weak. *Calcarea carb.*$^{mm}$ (F. C) once daily for seven days, and needed no more medicine.

(13) *Lycopodium*$^{mm}$ (F. C.) one dose, cured axillary perspiration *smelling of onions*, so bad that her mother could not use the same sponge.

(14) *Hepar.*—March 3d, 1882.—Miss W., aged seventeen. Never thoroughly strong since age of four. Twice has had pneumonia; the first time nine years ago with scarlatina; the second a year or two afterward. In the first attack was attended by the late Dr. Alabone (mongrel); in the second by an allopath. Two and a half years ago suffered from anæmia; consulted a dishonest mongrel who sneers at high potencies, and prescribes allopathic mixtures; afterward consulted an honest though mistaken allopath. Their treatment did no good; but she improved on going into the country.

*Present state.*—Tired, languid feeling, especially in morning. Pale. Appetite poor; dislikes fat; likes very strong tea. Unless she has a cold bath she catches cold in head, followed by tightness of chest. Feels worse alternate days. To diminish tea gradually and take pure solidified cacao; also to practice deep inhalations. *Hepar sulph.*$^{cm}$ (F.C.) once daily for fourteen days.

March 17th.—Decidedly better this last week. Less tired. More color in face. Appetite better, but still dislikes fat. *Hepar*$^{cm}$ (F. C.) alternate days for fourteen days.

Did not see her again, but subsequently she sent me a patient, who informed me that Miss W. had quite recovered.

I think it best to diminish tea, coffee, or alcohol *gradually*, not suddenly, if patients are very weak.

